# A Modified Bioceramic Sealer with Dual Antibacterial Mechanisms

**DOI:** 10.3390/bioengineering12070768

**Published:** 2025-07-16

**Authors:** Bashayer Baras, Amal Almohaimede, Yara Alshibani, Farah Alzahrani, Raseel Alageel, Michael D. Weir, Hockin H. K. Xu

**Affiliations:** 1 Department of Restorative Dental Science, College of Dentistry, King Saud University, Riyadh 11432, Saudi Arabia; bbaras@ksu.edu.sa; 2 Department of Restorative Dental Science, Endodontic Division, College of Dentistry, King Saud University, Riyadh 11432, Saudi Arabia; 3 College of Dentistry, King Saud University, Riyadh 11432, Saudi Arabia; yarashibani4@gmail.com (Y.A.); fh.a.alzahrani@gmail.com (F.A.); 4 Care Medical Hospital, Riyadh 14214, Saudi Arabia; rasel.alaqeel@gmail.com; 5 Department of Advanced Oral Sciences and Therapeutics, University of Maryland School of Dentistry, Baltimore, MD 21201, USA; michael.weir@umaryland.edu (M.D.W.); hxu2@umaryland.edu (H.H.K.X.); 6 Center for Stem Cell Biology & Regenerative Medicine, University of Maryland School of Medicine, Baltimore, MD 21201, USA; 7 Marlene and Stewart Greenebaum Cancer Center, University of Maryland School of Medicine, Baltimore, MD 21201, USA

**Keywords:** antibacterial agents, DMAHDM, endodontics, innovative materials, silver nanoparticles, physical properties

## Abstract

Continued efforts have been made to enhance the antibacterial properties of root canal sealers by adding antimicrobial agents to them. This study aims to investigate the antibacterial effect of 0.15% silver nanoparticles (NAg) and 5% dimethylaminohexadecyl methacrylate (DMAHDM) when added to EndoSequence Bioceramic (BC) sealer against *Enterococcus faecalis* (*E. faecalis*) biofilm and their impact on its physical properties (flowability and film thickness). Four root canal sealers were tested for flow and film thickness properties, as well as against antibiofilm of *E. faecalis*-impregnated dentin discs, as follows: group 1: EndoSequence BC sealer only; group 2: EndoSequence BC sealer + 0.15% NAg; group 3: EndoSequence BC sealer + 5% DMAHDM; and group 4: EndoSequence BC sealer + 0.15% NAg + 5% DMAHDM. The findings show that all groups had flow and film thickness values that were in accordance with the ISO requirements. Combining 0.15% NAg and 5% DMAHDM in EndoSequence significantly reduced colony-forming unit (CFU) counts by approximately 5 logs. The combination of NAg and DMAHDM offers a promising strategy for developing endodontic sealers with improved antimicrobial properties and acceptable physical performance.

## 1. Introduction

Successful endodontic therapy mainly aims to remove necrotic and inflamed pulpal tissues and eliminate or reduce bacterial biofilm so that the host can heal and regenerate damaged periapical tissues [1,2]. Adequate instrumentation followed by obturation of the root canal space ensures an impervious seal of the root canal system and successful treatment outcomes [3,4]. Failure often occurs due to incomplete eradication of root canal microbiota, which is caused by the complex anatomy of the root canal system and the resistance of root canal biofilms to irrigation solutions and intracanal medicaments [5,6].

Traditional instrumentation and irrigation techniques have previously shown their inability to eradicate bacteria from the root canal system [7]. Previous studies have shown that some endodontic bacteria can penetrate dentinal tubules to a depth of 200–1500 µm, making them harder to eliminate [8]. Enterococcus faecalis (*E. faecalis*) is one of the most common Gram-positive bacteria in the root canal system. It can invade dentinal tubules and withstand long periods of nutritional deprivation, making it challenging to eradicate. Previous studies found that in 23–77% of cases with failed endodontic treatments, *E. faecalis* was detected and frequently associated with secondary root canal infections [9,10,11].

Filling the root canal space with gutta-percha cones as the core filling material in combination with a sealer cement to create a hermetic seal of the root canal system remains the most widely accepted obturation technique in endodontics [12]. Root canal sealers fill the irregularities between the core material and the canal wall. According to Grossman, ideal root canal sealers should have antibacterial properties and be able to flow into complex root canal anatomy where it is difficult to access with irrigants and instruments [13]. Sealers are an important part of successful root canal therapy, and they have been continuously developed and modified to enhance their performance. Commercially available root canal sealers have shown some antibacterial effects [14]. However, these effects tend to reduce over time [15,16,17]. Continued efforts have been made to enhance the antibacterial properties by adding antimicrobial agents to root canal sealers, potentially promoting root canal therapy’s longevity and, subsequently, preventing tooth loss [18,19,20].

Silver, one of the most commonly used antimicrobial agents, showed strong antibacterial, antifungal, and antiviral effects when incorporated into various dental materials [21]. They have been recently reduced to nanoparticles of silver (NAg), with a particle size of 2.7 nm, which enables them to be added in low concentrations to avoid compromising the mechanical and physical properties of the material while still imparting potent antimicrobial effects [22,23]. The principal antibacterial mechanism of NAg involves inhibiting DNA replication, leading to cellular lysis and death [20].

Polymerizable quaternary ammonium salts (QASs) are another antibacterial agent widely used in dental materials, which showed long-term antibacterial effects [20,23]. It works by contact inhibition through the distortion of the electrical balance of the microbial cell membrane. When the positively charged (N+) site of a QAS comes into contact with the negatively charged bacterial wall, an increase in the bacterial osmotic pressure occurs, which causes the bacteria to explode under its osmotic pressure [23]. Previous studies have shown that a QAS’s antibacterial activity is influenced by the alkyl chain length; as the alkyl chain length of QAS increases from 3 to 16, the antibacterial properties significantly improve [24,25,26,27]. Dimethylaminohexadecyl methacrylate (DMAHDM) is a QAS with an alkyl chain length of 16 and has shown potent and long-term antibiofilm activity [20,27].

Nowadays, various bioceramic (BC) sealers are used in endodontics. EndoSequence BC sealer (Brasseler USA, Savannah, GA, USA) is a premixed bioceramic sealer that includes tricalcium silicate, dicalcium silicate, and calcium hydroxide as bioactive components; calcium phosphate monobasic; and thickening agents [28]. A previous study showed that the antimicrobial effect of EndoSequence BC sealer was greatly diminished at 7 days after mixing [29]. Another study showed that EndoSequence BC sealer showed the same antibacterial effect at incubation periods of 72 h and 7 days against *E. faecalis* [30].

Seung et al. examined the antibacterial properties of DMAHDM-containing epoxy-resin-based sealers. They combined DMAHDM and NAg into AH Plus root canal sealer (Dentsply Sirona, New York City, NY, USA), utilizing a modified direct contact test at 1, 7, and 14 days against *E. faecalis*. When adding 2.5% DMAHDM and 0.15% NAg, the sealer could sustain antibacterial activity for up to 14 days [31]. In a study conducted by Baras et al., they reported a four-fold reduction in magnitude in colony-forming units (CFU) against *E. faecalis* when adding 5% of DMAHDM into a methacrylate-based sealer [20]. In another study by Baras et al., they aimed to use different fractions of DMAHDM and NAg to develop a novel dual-cured sealer with a potent, long-lasting antibacterial effect and to evaluate how such additives affect the physical properties (flowability, film thickness, and sealing ability) of the sealer. Adding 5% DMAHDM and 0.15% of NAg positively enhanced the biofilm inhibition, as it reduced polysaccharide production and CFU counts produced by *E. faecalis* without negatively affecting the physical properties. It was found that this combination further reduced the CFU counts by six-fold compared with AH plus sealer [18]. Another study aimed to create a new root canal sealer by adding DMAHDM, NAg, and calcium phosphate nanoparticles (NACPs) to test the sealer’s antibacterial effect against *E. faecalis*, as well as its physical properties and remineralizing potential. The sealer showed significant inhibition of biofilms without adversely affecting the physical properties, and the sealer containing 30% NACPs regenerated dentin minerals and increased the dentin hardness to match that of sound dentin [32].

To our knowledge, the antibacterial effect of adding NAg and DMAHDM to a bioceramic sealer and their influence on the physical properties have never been reported. The objectives of the study were to modify a bioceramic root canal sealer by adding 5% DMAHDM and 0.15% NAg and determine the effects on the antibacterial and physical properties. The following hypotheses were tested: (1) incorporating NAg and DMAHDM into EndoSequence BC sealer would not negatively influence the sealer’s flow and film thickness properties; (2) incorporating NAg and DMAHDM into EndoSequence BC sealer would inhibit *E. faecalis* impregnated in dentin discs.

## 2. Materials and Methods

### 2.1. Preparation of the Modified Root Canal Sealer

EndoSequence BC (BC, Brasseler, Savannah, GA, USA) sealer was used as the sealer to which the bioactive agents, DMAHDM and NAg, were added. DMAHDM was synthesized using a modified Menschutkin reaction. In short, 10 mmol of 2-(dimethylamino) ethyl methacrylate (DMAEMA, Sigma-Aldrich, St. Louis, MO, USA), 10 mmol of 1-bromohexadecane (BHD; TCI America, Portland, OR, USA), and 3 g of ethanol were combined in a 20 mL scintillation vial to allow for a reaction between the tertiary amine group and the organo-halide. The vial was stirred at 70 °C for 24 h, and the solvent was allowed to evaporate. DMAHDM was incorporated at a 5% mass fraction, following previous studies, as this mass fraction produced the highest antibacterial effect without jeopardizing the material’s physical and mechanical properties [32]. To synthesize NAg, 0.1 g of silver 2-ethylhexanoate (Strem, Newburyport, MA, USA) was dissolved into 0.9 g of 2-(*tert*-butylamino) ethyl methacrylate (TBAEMA, Sigma-Aldrich) to allow for the maximum dissolution, as based on previous studies. This method resulted in the formation of 2.7 nm particles of silver that were stabilized in a polymeric matrix, which could allow for the continuous and regular release of silver particles instead of a burst release, which could result in their depletion after a short period of time. The NAg was incorporated into EndoSequence at a 0.15% mass fraction, following the results of previous studies [32]. A transmission electron microscope (TEM, Tecnai-T12, FEI, Hillsboro, OR, USA) was used to examine the NAg in the TBAEMA and confirm the particle size of the silver nanoparticles. An Ag-resin monomer was placed in the gap of a thin sheet of mica that was partially split. The resin in the mica was pressed to form a film and then light-cured. The resin film was used for a TEM examination.

The following four root canal sealers were tested for flow, film thickness, and antibiofilm properties:

- Group 1: EndoSequence BC sealer only;
- Group 2: EndoSequence BC sealer + 0.15% NAg;
- Group 3: EndoSequence BC sealer + 5% DMAHDM;
- Group 4: EndoSequence BC sealer + 0.15% NAg + 5% DMAHDM.

### 2.2. Flow Properties of the Sealers

Sealers should have a flow rate of ≥20 mm based on the International Standards Organization’s (ISO) 6876/2012 standards for root canal sealing materials [33]. The flow properties of the sealers were tested following the protocol recommended by the ISO 6876/2012 report. A volume of 0.1 mL of the mixed sealer paste was placed on a glass slab with dimensions of 40 × 40 × 5 mm using a graduated 1 mL syringe. An additional glass slab weighing approximately 20 g was then placed above the sealer, followed by a weight of approximately 100 g. This yielded a total mass of 120 ± 2 g. After 10 min, the weight was removed, and the diameter (mm) of the sealer was measured using a digital caliper (Mitutoyo, Tokyo, Japan). This was recorded as the flow rate of the sealer. Two readings were taken, and the average was calculated.

### 2.3. Film Thickness Properties of the Sealers

Root canal sealers applied in thick films are discouraged, as they are susceptible to degradation. Therefore, it is recommended by the ISO 6876/2012 standards for root canal sealing materials that root canal sealers have a film thickness of ≤50 µm [33]. The addition of bioactive agents can increase the film thickness; therefore, it was important to test the effect of adding DMAHDM and NAg to EndoSequence on the sealers’ film thickness. First, two glass slabs were combined, and their combined thickness was measured. A measured amount of the tested sealer was placed at the center of the glass slab, and the other glass slab was positioned on top. A loading device applied a load of 150 N on the top glass slab. After ten minutes of mixing and applying the load, the combined thickness of the two glass slabs with the film of sealer in between was measured using a micrometer (iGaging, Los Angeles, CA, USA) [33].

### 2.4. Antibiofilm Testing of E. faecalis-Impregnated Dentin Discs

Twenty single-rooted maxillary anterior teeth were transversely sectioned using a rotating diamond saw mounted on a slow-speed precision cutting machine (Buehler Isomet, IL, USA) with water cooling, approximately 3 mm below the cemento–enamel junction (CEJ), to obtain dentin sections measuring 1.5 ± 0.1 mm in thickness (n = 5). The canal lumens were enlarged using a water-cooled round bur (2.5 mm in diameter). All dentin sections were immersed in a 5.25% NaOCl solution, followed by 17% EDTA to remove the smear layer. Subsequently, the samples were immersed in brain heart infusion (BHI) broth (Sigma-Aldrich, Darmstadt, Germany) before being sterilized in an autoclave.

The dentin samples were then placed in microcentrifuge tubes containing 500 µL of an *E. faecalis* (ATCC 29212) suspension in BHI broth (Sigma-Aldrich, Darmstadt, Germany), adjusted to 10^7^ colony-forming units (CFU)/mL based on the bacterial growth curve using the optical density (OD) at 600 nm. The tubes underwent sequential centrifugation at 1400× *g*, 2000× *g*, 3600× *g*, and 5600× *g*, with each step repeated twice for 5 min. After each centrifugation cycle, the solution was replaced with a fresh bacterial suspension, following a previous study [34].

**Figure 1:**
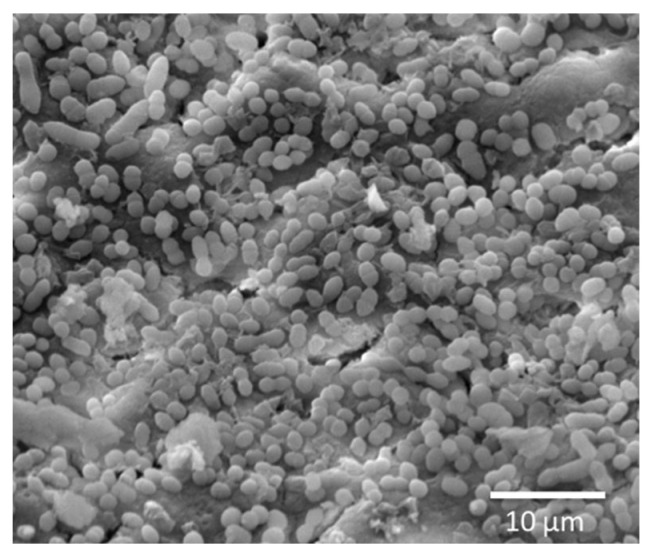
SEM image of *E. faecalis* colonization on the root canal wall.

The dentin samples were incubated in a 24-well plate containing 1 mL of BHI broth for 7 days to facilitate biofilm formation, with broth replacement every other day. After 7 days, the samples were transferred to a new 24-well plate and rinsed with sterile phosphate-buffered saline (PBS, Sigma-Aldrich, Darmstadt, Germany) [34,35,36]. During our pilot testing of the dentin impregnation biofilm model, scanning electron microscopy (SEM) images were taken of *E. faecalis* colonization on the root canal wall (Figure 1).

All root canal sealers were mixed and applied to the root canal space of bacteria-impregnated dentin samples, allowing them to set for 7 days at 37 °C and 100% relative humidity. To prevent excessive drying of dentin, a wet cotton pellet was placed on top of the dentin samples [32]. The samples were then transferred to microcentrifuge tubes containing 1 mL of PBS and subjected to sonication at 40 kHz for 5 min. This was followed by vortexing at maximum speed for 20 s using a vortex mixer (Vortex Maxi Mix II, Barnstead Thermolyne, Dubuque, IA, USA) to extract bacteria from the dentin samples.

**Figure 2:**
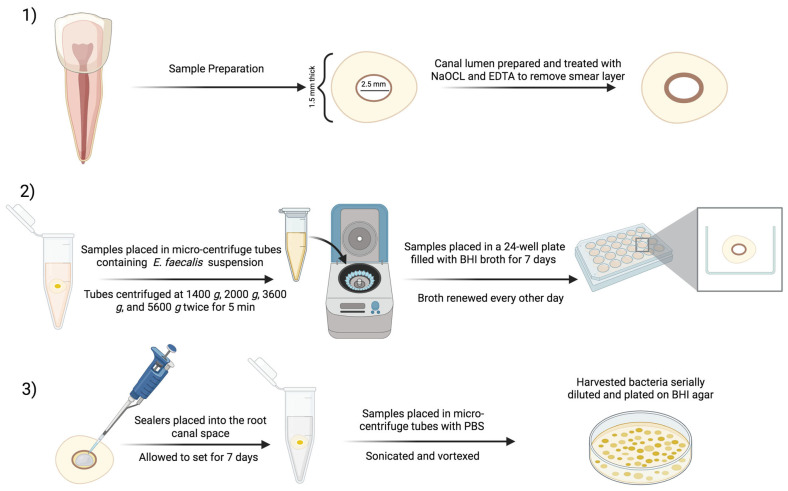
Schematic diagram of the experimental design of the antibiofilm testing of *E. faecalis*-impregnated dentin discs: (**1**) sample preparation and treatment; (**2**) *E. faecalis*-impregnation in dentin discs; (**3**) sealer placement and CFU. Created with Biorender.com.

The harvested bacteria were serially diluted, plated on BHI agar, and incubated for 48 h at 37 °C with 5% CO_2_ to determine the number of colony-forming units (CFU) (n = 5) (Figure 2).

## 3. Results

**Figure 3:**
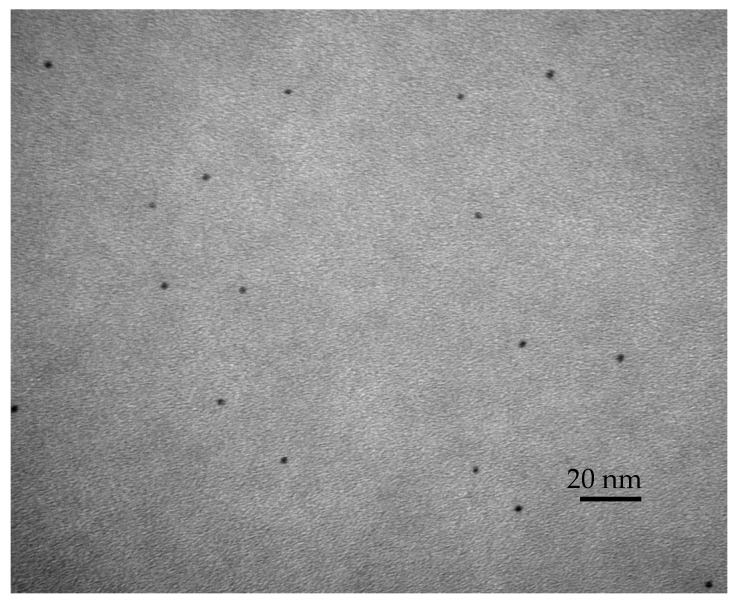
TEM image of silver nanoparticles. The NAg’s mean particle size was 2.7 ± 0.6 nm (mean ± sd; n = 100).

The transmission electron microscopy (TEM) examination revealed images of NAg with an average size of 2.7 nm (Figure 3), with the mean ± SD (n = 100). The NAg particles showed adequate dispersion without any nanoparticle agglomeration.

**Figure 4:**
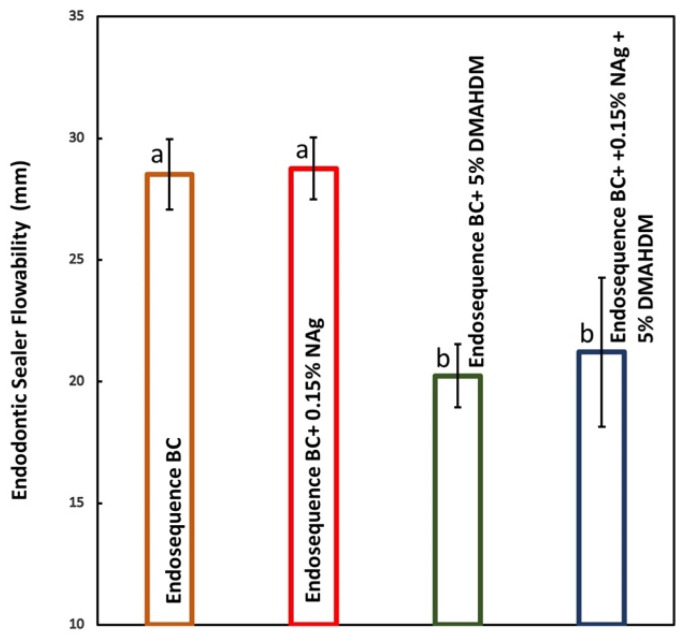
Endodontic sealers’ flow properties (mean ± sd; n = 3). Incorporating 0.15% NAg did not significantly affect the flowability of EndoSequence (*p* > 0.05). However, when 5% DMAHDM was added, whether alone or in combination with NAg, the flow properties were reduced (*p* < 0.05). All groups had flow values that were in accordance with the ISO specifications. Dissimilar letters indicate values that are significantly different from each other (*p* < 0.05).

The results of the endodontic sealers’ flow properties are demonstrated in Figure 4 (mean ± sd; n = 3). Incorporating NAg into EndoSequence did not influence the flow properties (*p* > 0.05). Adding DMAHDM alone or in combination with NAg significantly reduced the flowability of EndoSequence (*p* < 0.05). However, all sealers had flow properties ≥ 20 mm, which is the range recommended by the ISO standards.

**Figure 5:**
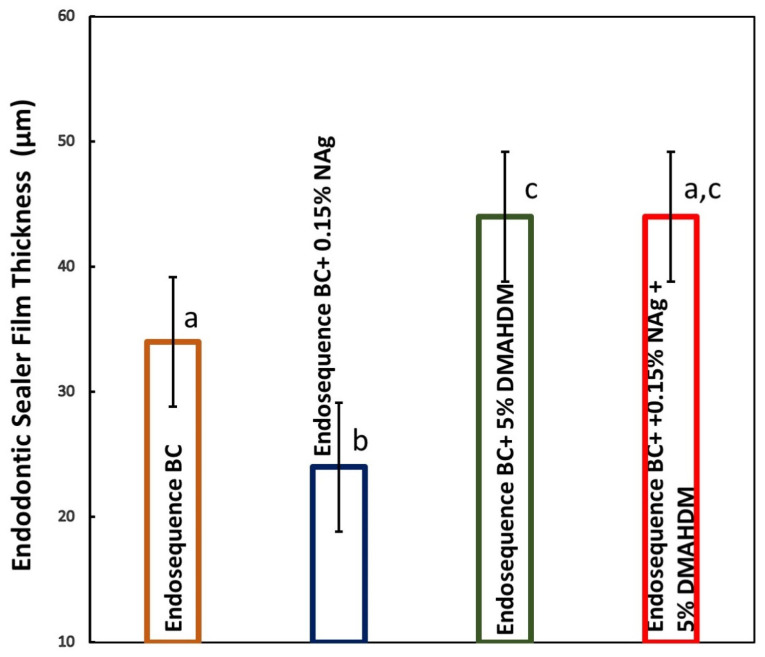
Endodontic sealers’ film thickness properties (mean ± sd; n = 5). Adding 5% DMAHDM and 0.15% NAg alone significantly influenced the film thickness of the EndoSequence sealer (*p* < 0.05). However, the group with DMAHDM + NAg had film thickness values like those of the control group (*p* > 0.05). All groups were in accordance with the ISO requirements (≤50 µm). Dissimilar letters indicate values that are significantly different from each other (*p* < 0.05).

The results of the film thickness properties of the endodontic sealers are plotted in Figure 5 (mean ± sd; n = 5). Incorporating DMAHDM and NAg alone significantly affected the film thickness of EndoSequence (*p* < 0.05). However, when both DMAHDM and NAg were incorporated together, the sealer produced film thickness values that were not significantly different from the control group (*p* > 0.05). All sealers had film thickness values that were in accordance with the ISO requirements (≤50 µm).

**Figure 6:**
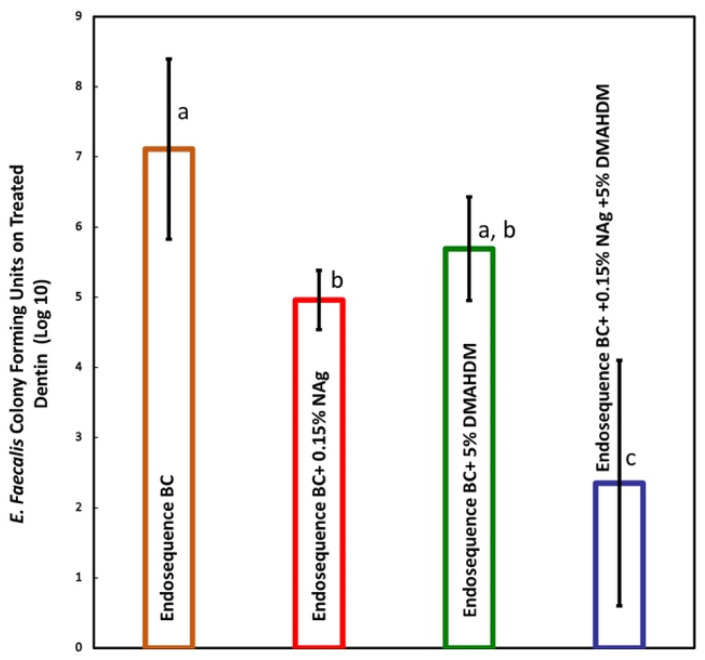
Biofilm CFU counts harvested from *E. faecalis*-impregnated dentin blocks treated with Endodontic sealers (mean ± sd; n = 5). The group with 0.15% NAg reduced CFU counts by nearly 2 logs when compared with the control group (*p* < 0.05). Biofilm CFU on blocks treated with 5% DMAHDM showed a CFU reduction of 1 and a half log (*p* > 0.05). Combining 0.15% NAg and 5% DMAHDM in EndoSequence resulted in a CFU reduction of approximately 5 logs (*p* < 0.05). Dissimilar letters indicate values that are significantly different from each other (*p* < 0.05).

The SEM image of *E. faecalis* colonization on the root canal wall can be seen in Figure 1. The 7-day biofilm CFU counts harvested from *E. faecalis*-impregnated dentin blocks treated with the modified sealers are presented in Figure 6 (mean ± sd; n = 5). Dentin blocks treated with EndoSequence BC+ 0.15% NAg reduced CFU counts by nearly 2 logs (*p* < 0.05). The addition of 5% DMAHDM alone resulted in a 1 and a half log reduction but was not statistically significant (*p* > 0.05). Combining 0.15% NAg and 5% DMAHDM into EndoSequence reduced CFU counts by approximately 5 logs (*p* < 0.05).

## 4. Discussion

The present study evaluated the effects of incorporating 0.15% NAg and 5% DMAHDM, individually and in combination, into EndoSequence BC sealer on its antibacterial efficacy against *Enterococcus faecalis* biofilm, as well as their impact on its key physical properties. 

Our findings demonstrate that the combination of NAg and DMAHDM had a significant synergistic effect on reducing *E. faecalis* biofilm, achieving approximately a 5-log reduction in CFU counts. This outcome is consistent with previous reports highlighting the enhanced antimicrobial action when quaternary ammonium compounds were combined with silver nanoparticles [18,20,31,32]. NAg alone produced a significant, though less pronounced, antibacterial effect, while DMAHDM alone achieved a modest, statistically nonsignificant reduction. These findings support the hypothesis that combining antibacterial agents can yield enhanced bioactivity through complementary mechanisms, as follows: NAg’s bacterial membrane disruption and DMAHDM’s contact-killing capability [37].

The use of 5% DMAHDM was associated with an increased surface charge density, which could explain the higher antibacterial activity against multispecies biofilm compared with the 3% formulation [38,39]. NAg is considered a releasing antibacterial agent with broad-spectrum outcomes [40,41]. The principal antibacterial mechanism of NAg involves the release of silver ions, which disrupt the activity of essential bacterial enzymes, thereby inhibiting DNA replication and leading to cellular lysis and death. Moreover, NAg exhibits a long-range antimicrobial effect, enabling it to eradicate bacteria located at a distance from the material surface [20,42,43]. Silver nanoparticle incorporation into root canal sealers confers a distinct advantage over micro-sized particles, attributable to their nanoscale characteristics and reduced particle size, which facilitate enhanced antimicrobial efficacy even at lower filler concentrations [20]. Baras et al. showed that the use of 0.15% NAg in a methacrylate-resin-based experimental sealer resulted in a 2-log CFU reduction in *E. faecalis* biofilm on saliva-coated resin discs compared with the experimental control group without NAg, and it was able to significantly reduce biofilm polysaccharide production [18], and when mixed with 5% DMAHDM, it resulted in a 6-log CFU reduction in *E. faecalis* biofilm [18]. TBAEMA was used because it improves the solubility of silver ions by forming Ag-N coordination bonds with Ag ions, thereby facilitating the Ag salt’s dissolution in the resin solution. The effect of TBAEMA and different concentrations of silver salt on the material’s antibacterial and mechanical properties has previously been investigated [22].

Regarding the physical properties, the addition of NAg had no significant effect on the sealer’s flowability, aligning with the previous literature showing that low concentrations of silver nanoparticles do not compromise the sealer’s rheology [18,44]. However, the addition of DMAHDM, whether alone or combined with NAg, resulted in a statistically significant reduction in flowability. On the other hand, the addition of NAg or DMAHDM significantly affected the sealer’s film thickness. A previous study showed that the addition of DMAHDM to AH Plus and bioceramic (BC) endodontic sealers increased their film thickness [39], which is in accordance with the results of our study. On the other hand, other studies showed that the addition of NAg did not negatively affect the film thickness [18,32]. However, combining NAg and DMAHDM did not adversely affect the film thickness, which is in accordance with previously published results [18,32].

However, all sealers had flow properties ≥ 20 mm, which falls within the range recommended by the ISO standards, indicating that the modified sealers remain clinically acceptable for filling regions that are challenging to reach. This is in accordance with previously reported studies [18,31,39]. Similarly, film thickness was unaffected negatively by any modification, with no significant differences observed across groups, suggesting that the additives do not compromise the sealer’s ability to form an adequate seal, which is also in accordance with previously reported studies [18,39].

Despite the promising results, this study has some limitations that need to be considered in future research. First, using a single-species biofilm model does not adequately replicate the complexity of clinical conditions that contain multiple-species biofilm. Additionally, the biofilm used in this study was relatively young and lacked the maturity required to accurately reflect the aggressiveness of endodontic infections. Moreover, the model used in this study is an in vitro model, which might not mimic the real, challenging clinical oral environment. Second, further investigations, using in vitro and ex vivo models, into other physical, mechanical, and biological properties, such as pH, setting time, dimensional stability, radiopacity, solubility, adhesive strength to dentin, and biocompatibility with surrounding tissues to promote healing, are warranted.

Overall, the results suggest that incorporating NAg and DMAHDM into bioceramic sealers enhances the antibacterial performance significantly while preserving the physical properties required for clinical use. These findings support the development of dual-functionalized sealers for improved long-term endodontic outcomes. 

## 5. Conclusions

The incorporation of 0.15% NAg and 5% DMAHDM into EndoSequence BC sealer demonstrates a strong synergistic antibacterial effect against *E. faecalis* biofilms. Importantly, these modifications did not adversely affect the sealer’s film thickness and maintained the flowability within ISO standards.

## Data Availability

The raw data supporting the conclusions of this article will be made available by the authors upon request.
